# Breast tuberculosis in men: A systematic review

**DOI:** 10.1371/journal.pone.0194766

**Published:** 2018-04-03

**Authors:** GianLuca Quaglio, Damiano Pizzol, Anna Bortolani, Fabio Manenti, Petros Isaakidis, Giovanni Putoto, Piero L. Olliaro

**Affiliations:** 1 European Parliamentary Research Services (EPRS), European Parliament, Brussels, Belgium; 2 Department of Internal Medicine, Verona University Hospital, Verona, Italy; 3 Doctors with Africa CUAMM, Operational Research Unit, Beira, Mozambique; 4 Doctors with Africa CUAMM, Operational Research Unit, Padua, Italy; 5 Médecins Sans Frontières, Southern Africa Medical Unit (SAMU), Cape Town, South Africa; 6 Special Programme for Research and Training in Tropical Diseases, World Health Organization (WHO/TDR), Geneva, Switzerland; 7 Centre for Tropical Medicine and Global Health, Nuffield Department of Medicine, University of Oxford, Oxford, United Kingdom; Public Library of Science, UNITED KINGDOM

## Abstract

**Setting:**

Breast tuberculosis in male is a rarely reported and poorly described condition.

**Objective:**

To quantify the number of breast tuberculosis in men, to describe clinical presentation and to present the diagnostic and therapeutic procedures applied.

**Design:**

A systematic review of the literature including reports published in English, Spanish and French until December 2017.

**Results:**

The search yielded 26 cases of male breast tuberculosis, median age 56.5 years. Most presented with an isolated breast lump (89%), associated with axillary lymphadenitis (27.8%) and skin inflammation (33.3%). The most common constitutional symptoms were pain (64.7%) and fever (35.3%). Fine-needle aspiration cytology and culture were the most common diagnostic modality (61.5%). Standard anti-tuberculosis regimen was the main treatment, alone or accompanied or preceded by incision and drainage.

**Conclusions:**

The risk of breast tuberculosis in men appears to be low, but the condition can be difficult to diagnose and the diagnostic delays can be long. Overall prognosis is good following standard anti-tuberculosis regimen with or without incision/drainage.

## Introduction

Tuberculosis (TB) is a chronic granulomatous inflammation usually involving the lung parenchyma and hilar lymph nodes. Extra-pulmonary involvement is seen in ~20% of all TB cases [1]. TB of the breast is an uncommon disease, particularly in men [2,3]. The first case of breast TB was reported in a woman in 1829 [4], but the first detailed description of the disease was only made by the end of the 19th Century [5, 6]. The first case of breast TB in a man was reported about a century later in 1927^7^, and by 1945 there were only 21 known cases of breast TB in men [7–10]. It is generally believed that the infection of the breast is usually secondary to tuberculous foci elsewhere in the body, which may or may not be clinically apparent [11, 12]. There are no well-defined clinical features of male breast TB, which may be confused with other clinical conditions, such as gynecomastia and breast carcinoma [13]. Therefore, TB of the breast can be difficult to diagnose and the diagnostic delay can amount to several months. To our knowledge, one systematic review has been published to-date on breast TB in man [3]. The review identified 24 cases, which presented mostly with an isolated breast lump; constitutional symptoms were rare; fine needle aspiration cytology (FNAC) was the most common diagnostic modality. However the review had limitations: it covered only the English literature, 16 of these cases were from mixed female and male series (where information on the male cases were challenging to extrapolate), and only 8 described as single case report. Therefore, we aimed to update and expand the existing evidence-base by systematically reviewing the English, Spanish and French literature for single-case reports and case-series about risk factors, clinical appearance of lesion and clinical presentations, constitutional symptoms, diagnostic procedures; and anti-tubercular and surgical treatments. of breast TB in men.

## Methods

### Search strategy

**Fig 1:**
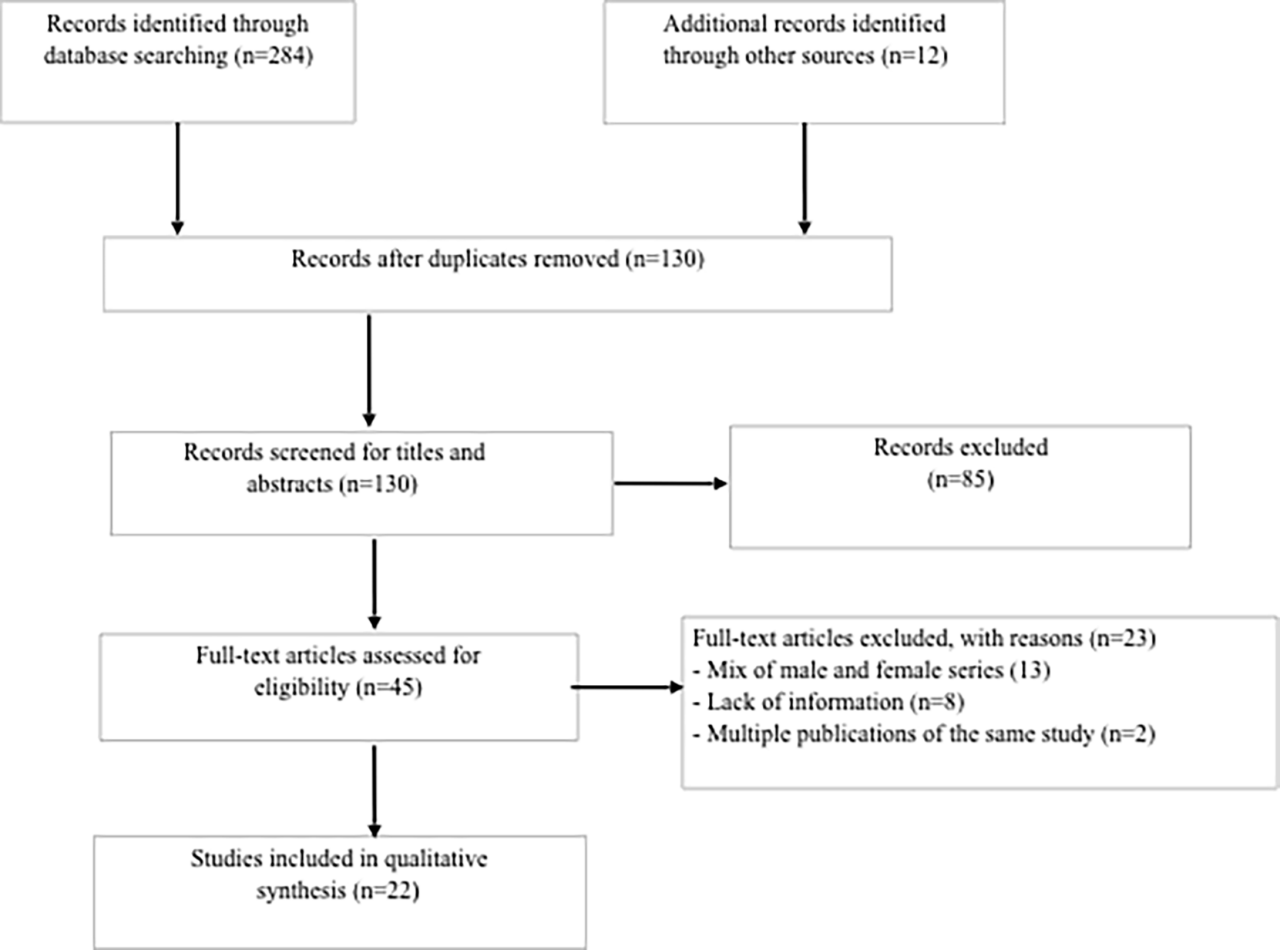
Study selection flowchart.

Searches were undertaken in PubMed, Embase and Web of Science. A search strategy was developed using a combination of free text and controlled vocabulary terms and adapted for each database. We used the following search strategy: (tuberculosis OR TB) AND (breast OR mammary OR mastitis) AND (men OR male). We included reports of studies published in English, Spanish and French until December 2017. Additional studies were identified by contacting the authors and by searching the reference lists of primary studies. The process of study selection is summarized in Fig 1. Titles and abstracts identified through the searches were reviewed independently by two reviewers (GQ and DP). In case of duplicate publications, the most recent publication which reported full data was included. Full text copies of the selected studies were retrieved and independently reviewed against the inclusion criteria by two reviewers from a team of three (DP, GP, GQ). Outcome measures included: i) risk factors for breast TB; ii) clinical appearance of lesion and clinical presentations; iii) constitutional symptoms; iv) diagnostic procedures; and v) anti-tubercular and surgical treatments.

### Data extractions and analysis

Each of the included studies was coded with a pre-formulated rating sheet with relevant data extracted and recorded by two reviewers. The data extracted included: name of the first author, year of publication, country where the study was conducted, general participant characteristics (age and gender) risk factors (HIV+, previous history of TB), breast affected (right, left), type of lesion (lump, abscess, disseminated), clinical presentations (sinus or fistula, skin ulceration, nipple retraction, discharging sinus), constitutional symptoms (fever, decreased appetite, decreased weight, pain), duration of symptoms, previous empirical antibiotic treatment, previous steroids treatment, previous chemotherapy, chest X-ray results, diagnostic procedure (biopsy, FNAC, Ziehl-Neelsen, culture, polymerase chain reaction (PCR), surgical procedure and type and duration of anti-tubercular treatment. Since this review identified mainly case-reports and a limited number of small case-series, a meta-analysis was not considered relevant.

### Publication bias

This is a systematic review of non-analytical studies i.e. case-series and cases-reports and the assessment of publication bias might not be practically applicable [14].

### Quality evaluation of studies

The Grading of Recommendations Assessment, Development, and Evaluation (GRADE) and the approach by the Agency for Healthcare Research and Quality (AHRQ), are not suitable to be used for our systematic review of non-analytical studies. For this reason we have not performed a formal evaluation of the quality of the included studies [14].

## Results

### Study selection and characteristic of included studies

We identified 284 potentially eligible studies from the database searches, and 12 studies screening of bibliographies and by contacting the authors. We removed duplicates and screened the titles and abstracts of the remaining 130 records: 45 publications were selected for full-text screening, from which 22 articles were included in the final analysis (Fig 1). Thirteen articles reported mixed males and females cases, from which it was not possible to obtain specific information on individual male cases, and therefore could not be included in the analysis [15–27] (Table 1).

**Table 1:** Articles reported mixed males and females cases.

Author	Reference	Year	Country	No. of male cases
Shinde	15	1995	India	3/100
Kakkar	16	2000	India	6/160
Khanna	17	2002	India	2/52
Puneet	18	2005	India	1/42
Bani-Hani	19	2005	Jordan	1/9
Harris	20	2006	India	1/38
Metha	21	2010	India	1/63
Lin	22	2010	Taiwan	1/26
Meerkotter	23	2011	South Africa	1/21
Chandanwale	24	2012	India	1/11
Ramaema	25	2015	South Africa	1/65
Raza	26	2016	India	1/9
Darré	27	2017	Togo	2/28

Other studies were excluded for lack of information or multiple publications of the same study (Fig 1). These 22 articles reported on 26 cases of male breast TB [3, 28–48]; one article included 3 cases [37], two reported 2 cases [28, 39] and 19 are single case reports [3, 29–36, 38, 40–48] (Table 2). The information collected on different variables differ from study to study as the data were not fully available in the case reports collected.

**Table 2:** Table summary of 26 reported cases of breast TB in men.

Author, year and ref.	Country	Age	Duration of symptoms (weeks)	Type of lesion (localization)	Signs	Symptoms	Diagnosis	Medical therapy (months)	Surgery
Wilson, 1990 [28]	USA	83	130	Disseminated lesion (L)	Discharge, lymphadenopathy	Pain	X-ray-, culture+, AFB-	OATT (24)	Drainage
Wilson, 1990 [28]	USA	66	3	Lump (L)	None	None	X-ray*, biopsy-, culture+, AFB-	OATT (18)	Excision
Jaideep, 1997 [29]	India	43	12	Lump (R)	Nipple retraction	Pain	X-ray-, FNAC+	ATT (6)	Excision
Thompson, 1997 [30]	USA	58	4	Lump (R)	Skin inflammation	Weight loss, fever, cough	X-ray-, FNAC+, culture+, AFB+	U	Excision and drainage
Reyes, 1999 [31]	USA	68	4	Lump (R)	Skin inflammation	Weight loss, fever, cough	X-ray-, FNAC+, culture+, AFB+	U	Drainage
Luna, 2000 [32]	Spain	17	8	Lump (L)	U	U	Biopsy+, culture+, AFB-	OATT (6)	Drainage
Gupta, 2002 [33]	India	25	U	Lump (R)	U	Pain, fever	FNAC+	ATT (6)	None
Bani-Hani, 2005 [34]	Jordan	68	U	Lump (L)	U	U	X-ray*, biopsy+	ATT (6)	Excision
Winzer, 2005 [35]	Germany	53	U	Lump (R)	U	U	X-ray-, culture-, PCR+	U	Excision
Marie, 2007 [36]	France	63	12	Lump (R)	U	U	Biopsy+,Culture+, AFB+, PCR+	OATT (U)	Drainage
Reyes, 2007 [37]	USA	68	U	Disseminated lesion (R)	Skin inflammation	Fever, chills, poor appetite, nonproductive cough	FNAC+, AFB+culture+	U	Drainage
Reyes, 2007 [37]	USA	59	U	Lump (U)	Skin ulceration, discharge	U	FNAC+, AFB+	U	Drainage
Reyes, 2007 [37]	USA	29	U	Lump (U)	Skin ulceration, discharge	U	FNAC+, AFB+	U	Drainage
Ursavas, 2007 [38]	Turkey	41	8	Lump (R)	None	Pain	X-ray-, FNAC+, culture+, AFB+	ATT (9)	None
Luh, 2007 [39]	China	92	8	Lump (R)	Skin inflammation, discharge	Pain, anorexia, body weight loss	X-ray-, culture-, FNAC-, biopsy+	U (6)	Excision
Luh, 2007 [39]	China	80	U	Lump (L)	Skin inflammation,discharge	U	FNAC-, biopsy+	U (6)	Excision
Rajagopala, 2008 [3]	India	25	U	Lump (R)	None	Pain	X-ray+, FNAC+, AFB+	ATT (6)	None
Moujahid, 2011 [40]	Morocco	50	U	Lump and abscess (L)	Skin ulceration, discharge, lymphadenopathy	Pain, weight loss, fever	X-ray-, biopsy+, culture+	ATT (9)	Drainage
Cantisani, 2013 [41]	Italy	28	U	Lump (R)	U	Pain	X-ray-, FNAC+, culture+, AFB+	ATT (5)	None
Ssen, 2013 [42]	Madagascar	23	20	Lump (R)	Lymphadenopathy	None	X-ray-, biopsy+	ATT (8)	Excision
Mahajan, 2014 [43]	India	20	12	Lump (L)	Lymphadenopathy	Pain	X-ray-, FNAC+, culture+, AFB+	ATT (6)	None
El Hammoumi, 2014 [44]	Morocco	55	U	Lump (L)	Skin inflammation, lymphadenopathy	U	X-ray-, FNAC+, biopsy+, culture+	ATT (6)	Drainage
Prakash, 2015 [45]	India	60	260	Lump (R)	U	U	FNAC+, biopsy+, AFB-	ATT (U)	Excision
Khaparde, 2015 [46]	India	60	8	Lump (R)	None	Pain	FNAC-, culture+, AFB+, biopsy+	ATT (6)	Excision
Brown, 2016 [47]	UK	44	U	Abscess (L)	U	None	X-ray+, culture+, AFB-, PCR+	ATT (6)	Drainage
Orerah, 2016 [48]	Kenya	70	24	Lump (L)	None	Pain, weight loss, fever, night sweats, loss of appetite	PCR+	ATT	None

L = Left; R = Right; ATT = Antitubercular Therapy; OATT = Others Antitubercular Therapy; FNAC = Fine-Needle Aspiration Cytology; AFB = Acid-Fast Bacilli; PCR = Polymerase Chain Reaction; U = Unknown; X-ray* = Previous TB.

### Geographic distribution and clinical characteristics

Cases were from Asia (n = 8 (30.8%), of which 6 from India and 2 from China), the USA (n = 7, 26.9%), Europe (n = 5 (19.2%) one each in Spain, Italy, Germany, UK and France), Africa (n = 4 (15.4%) from Morocco and Madagascar) and the Middle East (n = 2 (7.7%) from Jordan and Turkey). The median age was 56.5 years (range 17–92).

Location was reported as unilateral in all 26 cases, 14 of which in the right breast, 10 in the left breast and 2 not reported. Clinical presentation as a lump was described in 23 cases (89%), of which one case was accompanied by an abscess; the others were one case of isolated breast abscess and two cases of disseminated lesions. Associated findings were skin inflammation (alone or associated with discharge and/or lymphadenopathy) in 6 cases (33.3%), axillary lymphadenopathy (either alone or associated with discharge and/or skin inflammation), in 5 cases (27.8%), skin ulceration (along with discharge and/or lymphadenopathy) in 3 (16.7%); there was one case of nipple retraction and sinus or fistula (5.5%); no associated findings were reported in 8 cases. General symptoms were unknown in 9 cases (34.6%). Three patients did not report any accompanying symptoms. In the remaining 14 cases, pain (alone or with fever and/or weight loss), was reported by 11 subjects (64.7%) fever by 6, weight loss by 5 and cough by 3, all with other symptoms.

### Prior history and diagnosis

The duration of symptoms before seeking medical care was reported for 13 cases, as was highly variable, ranging from 3 to 260 weeks (median 18 weeks). Earlier history of TB was reported for 13 subjects, 5 of them (38.5%) had previous history of the disease. HIV status was reported for three cases, all negative. Information relating to previous antibiotic treatment was reported for 14 subjects, of whom three had received empirical antibiotic treatment before the final diagnosis of breast TB. In the clinical history of the 26 cases described, there was no information related to previous steroid treatments. One patient received chemotherapy for a lymphoma 5 years before developing breast TB [46].

The diagnostic procedure was documented in all 26 cases. Different diagnostic methods were combined. A chest X-ray was performed in 16 cases: it was negative in 12 (75%), showed signs of active TB in 2 and previous TB in another 2. FNAC was carried out in 16 cases all negative except 3 positive. Ziehl-Neelsen acid-fast bacilli (AFB) staining was performed in 16 patients, of which 11 were positive. Culture was performed in 16 cases, all but 2 positive. Biopsy was positive in 8 out 9 cases. PCR was performed in 4 cases, all of which positive. Using culture or PCR as the gold standard, the sensitivity of AFB was 66.7% (8/12 culture- and/or PCR positive cases; the specificity could not be determined as there were no concomitant culture-negative cases tested with AFB); all six cases tested with FNAC and culture were positive on both. The only negative culture was positive by PCR.

### Medical treatment and surgical treatment

Twenty patients (76.9%) received surgical treatment (10 drainage, 9 excision, one both drainage and excision). Eight subjects (30.8%) received surgical treatment without systemic medication. Six patients (23.1%) received systemic medication without surgical treatment. Twelve subjects (46.1%) received a combination of surgical and medical treatment. The standard anti-tuberculosis regimen (four-drug intensive phase with isoniazid, rifampicin, pyrazinamide and ethambutol, followed by a two-drug continuation phase with isoniazid and rifampicin) was the main treatment, used in 18 (76%) patients. Twenty-four subjects reported complete remission, and no cases of recurrences were described. Information on the follow up was missing for two cases. The follow up period (reported for ten cases) was 49.8 + 50.3 months.

## Discussion

This systematic review identified 26 cases of breast TB in men reported in the literature. It provides an up-to-date description of the clinical presentation, diagnostic and therapeutic procedures of breast TB in men, though it cannot conclude whether this condition is actually rare or under-diagnosed. Previous studies of breast TB (see Table 1) have identified low numbers of cases altogether, with women being at much higher risk with ratios of 8 women to 1 man reported in Tunisia (1980–2001) [19]; 65:1 in South Africa (2000–2013) [25]; 63:1 (1992–2008) [21] and 52:2 in India (1986–2000) [17]; 30:0 in Pakistan (1999–2007) [49]; 29:0 in Peru (2002–2011) [50]. In addition, in some series of male breast lesions, TB was not represented: in a series of 113 men with breast lesions over 15 years, 93% had gynecomastia, 2 patients had primary breast cancer and one had metastatic lymphoma [13]. In another series of 241 males over 8.5 years no case of breast TB was reported [51].

There is no known risk factor for male breast TB. It is generally though that in pregnant and lactating women the increased vascularity of the breast with dilated ducts can predispose to tubercular infection. Pregnancy suppresses the T-helper 1 pro-inflammatory response, which may increase susceptibility to new infection [52]. HIV coinfection exposes people to an increased risk for primary or reactivation TB and for second episodes of TB from exogenous reinfection [53, 54]. HIV has been considered as potential risk factor for breast TB, in both genders, however, this is not supported by evidence: no case of breast TB in women was found to be HIV-positive in a prospective series carried out in Peru of 28 female cases [55] or in a retrospective series of 20 female cases [56]. Breast TB as a presenting manifestation of HIV is extremely rare [57]; although in the present study HIV status was described in only 3 males, none was positive.

The clinical presentation of breast TB in men is poorly described in the literature, and important elements are not uniformly reported, or not reported at all. The combination of a-specific signs and symptoms, the challenges of diagnosing breast TB especially in resource-constrained settings, and lack of awareness of the condition, both by the patient and the health provider, leads to significant diagnostic delay: the median delay in diagnosis was 18 weeks in this review. The main differential diagnosis to be considered are, among others, gynecomastia [13], breast cancer [58], non-tubercular granulomatous mastitis [59, 60], atypical mycobacterial mastitis [61], plasma cell mastitis [62], ethionamide and isoniazid associated gynecomastia [63, 64].

Breast TB is often difficult to diagnose in both genders. Computer tomography scan, magnetic resonance, mammography and ultrasound can provide useful information, particularly on the extent of the disease. However, none of these image findings are specific for breast TB. Coexistent pulmonary TB may be suggestive of breast TB, but active disease is often absent; in this review, chest X-ray was reported in 16 cases, with only two cases positive for active TB. FNAC, culture, and biopsies have been used for diagnosis [3, 43, 44]. Culture might be seen as the gold standard, though its complexity, time-lag between sampling and obtaining a result, and possibility of false-negative results in paucibacillary specimens makes it unsuited to large-scale use [47]. However, culture performed well in the present review, with all but one of the cultures done testing positive. The yield of FNAC, which detects the presence of epithelioid cell granulomas and necrosis, is variable. In the present and past reviews [3], it was the most common diagnostic modality for breast TB. Here, it proved highly sensitive, as all specimens tested for both FNAC and culture were positive. The ongoing roll-out of PCR closer to the point of care may increase the utility of this method [65]. Here, it was used in only four cases, too few to draw any conclusion, except that PCR was positive in the single culture-negative case. This review suggests that diagnosing breast TB requires a combination of clinical, history and radiological findings complemented with when possible two diagnostic techniques, including FNAC with AFB staining, PCR or culture, whenever possible.

Breast TB overall appears to have a good prognosis, though no specific guidelines are available for the treatment of breast TB, whether in men or women. The optimum duration of therapy is unclear, and objective criteria for assessing response are lacking [3]. A standard anti-TB regimen (four-drug two-month intensive phase followed by a two-drug four-month continuation phase), often accompanied (or preceded) by incision and drainage or lump excision appears to achieve satisfactory responses. Twelve subjects received surgical treatment. However, it is not possible to determine to which extent these procedures were performed as an initial step towards establishing a diagnosis, rather than being part of the treatment plan.

In conclusion, breast TB in men is a rarely reported entity, even in high TB burden countries. Since the clinical features are not well defined, TB of the breast can be difficult to diagnose and the diagnostic delays can be long. The most common presentation is a lump with skin inflammation, pain and the involvement of axillary lymph nodes. FNAC, culture, and biopsies have been used for diagnosis. No specific treatment guidelines are available. Standard anti-TB drugs appear to achieve satisfactory responses and overall prognosis is good.

## Supporting information

S1 PRISMA Checklist (PDF)
